# Towards maximising information extraction from rodent models of ocular disease

**DOI:** 10.1038/cddis.2016.174

**Published:** 2016-06-23

**Authors:** B M Davis, L Guo, J Brenton, L Langley, E M Normando, M F Cordeiro

**Affiliations:** 1 Glaucoma and Retinal Neurodegeneration Research, Visual Neuroscience, UCL Institute of Ophthalmology, London, UK; 2 Western Eye Hospital, Imperial College Healthcare Trust, London, UK

Loss of retinal ganglion cells (RGCs) has a central role in retinal disorders including glaucoma, which result in progressive vision loss over time. The global glaucoma prevalence is currently estimated to be 3.54% of the population aged between 40 and 80 years.^1^ Currently, raised intraocular pressure (IOP) presents the only modifiable risk factor. However, some glaucoma patients continue to lose vision despite having well controlled IOPs.^2^ This has led to a search for alternative strategies to promote RGC preservation.^3^ Experimental glaucoma models and whole-retinal mounts have proven a useful *ex vivo* tool for the assessment of potential new treatments using well-established protocols for labelling RGCs, including using nuclear-restricted transcription factor brain-specific homeobox/POU domain protein 3A (Brn-3A).

Animal models of ocular disease have made an invaluable contribution to the development of new therapies, dramatically enhancing the quality of life of people with sight-threatening diseases.^4^ Increasing scrutiny of the scientific, ethical and economic use of procedures involving animals, however, has resulted in growing pressures ‘to reduce the sum total of pain and fear inflicted on animals by man' via the 3R's approach.^2^ In this context, reduction is defined by Russell and Burch's as a ‘Reduction in the number of animals used to obtain information of a given amount or precision'.^5^ Overzealous efforts to achieve this goal by simply minimising the number of animals used in experiments (for cost or ethical reasons) can, however, undermine study reliability and therefore result in unnecessary distress.^6^ Although the appropriate use of statistical methods to assess study power can be used to indicate the minimum number of animals required to conduct an experiment with the required power, a lack of preliminary data owing to variability in the implementation of models or experimental end points can impede their use.^5, 7^ One solution to this problem is to increase the amount of useful information extracted from each laboratory animal used in an experiment.

RGC quantification from Brn-3A-labelled retinal whole mounts is frequently achieved by sampling regions for manual counting. Although this technique provides an improvement on assessing RGC health from histological cross-sections (where typically as little as 0.1% of the total RGC population are evaluated), it remains susceptible to error, both from inter- and intra-operator variability and variation of RGC density across a retina (i.e., a threefold difference in RGC density is typically reported between central and peripheral rodent retina). In recent years, the development of automated whole-retinal measures of RGC density has sought to overcome limitations of the sampling approach, typically reducing each whole-retinal mount into a single mean RGC density data point.^8^ As typical healthy adult rat retina contains between 80 000 and 100 000 RGCs, a large amount of potentially useful information is discarded by only reporting changes as a single mean RGC density.

To resolve this problem, we^9^ describe a simple technique to extract greater information from RGC populations in retinal whole mounts in two established rodent models of optic neuropathy: the partial optic nerve transection (pONT)^10^ and Morrison's ocular hypertension (OHT) models.^11^ Each of these models are used to assess secondary degeneration of RGCs, which is thought to have a role in diseases such as glaucoma. Improving our understanding of the spatial-temporal pattern of RGC loss using these models can be used to provide useful insights regarding the disease mechanism and the activity of therapeutic interventions (Figure 1).

Using this technique, we demonstrate the spatiotemporal pattern of RGC loss in each model to a greater resolution than that has previously been achieved by dividing the retinal area into a series of 60 nonoverlapping segments from which RGC density, nearest neighbour distance and regularity index were calculated. In addition, by monitoring the longitudinal rate of RGC loss in each model, the proportion of primary and secondary degeneration in each retinal segment could be estimated. To evaluate the validity of this approach, the technique was first used to investigate the extent of RGC density loss and the distribution of primary and secondary degeneration events in pONT. Results obtained using our technique were in broad agreement with the existing literature with a greater proportion of primary degeneration and RGC loss reported in the superior retinal quadrant.^12^ The technique was next applied to the less well-described OHT model. The greatest loss of RGCs was observed along the superior-inferior axis, a result which shows a striking similarity to the bow-tie pattern of retinal nerve fibre layer injury reported in clinical glaucoma patients.^13^ This is a surprising result as the rat possesses a relatively simple lamina cribrosa structure relative to humans, which has been suggested to be responsible for this pattern of cell loss.^13, 14^ Having established the natural history of pONT and OHT models, this study provides sufficient preliminary data to calculate the statistical power of future studies aimed at investigating the efficacy of therapeutic interventions.

The retina offers the unique opportunity to non-invasively image structures of the central nervous system and comprises a well-established anatomy and simple structure compared to the brain. This, combined with the growing recognition that the retina also undergoes marked changes in neurodegenerative disorders makes retinal analysis an attractive tool to investigate neurodegenerative processes.^15^ A better understanding of ocular changes during neurodegenerative diseases is anticipated to yield novel early diagnostic techniques for disorders such as Alzheimer's Disease, which are urgently required.^15^ To this end, the analysis techniques described in this work with RGCs can readily be combined with well-established specific markers for other anatomic features including; glial, vasculature and retinal barrier function in order to provide a surrogate method to evaluate cellular pathology of neurodegenerative disease in the brain. This is significant as not only could this facilitate the development of novel diagnostic tests for neurodegenerative disease, but could also enhance our understanding of the mechanism of neurodegenerative disease progression and provide a much needed time frame for early changes in the disease process.

In summary, the development of techniques such as those described by our recent paper contribute to a growing body of work that seeks to maximise the amount of information that can be extracted from each laboratory animal and provide tools to increase the uniformity of techniques employed by groups using animals in research.

## Figures and Tables

**Figure 1:**
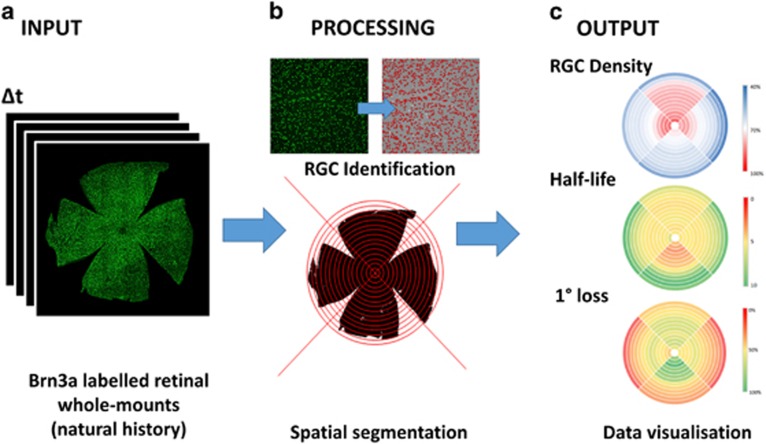
Maximising the extraction of information from retinal whole-mounts. (**a**) Brn-3a retinal whole-mounts were obtained at different timeponts from established rodent models of optic neuropathy. (**b**) The algorithm we developed^9^ first segmented the RGC population from these whole-mounts before spatially segmenting the population into a series 60 of non-overlapping sectors defined relative to the position of the optic nerve head. (**c**) Assessment of longitudinal changes across the natural history of each model permitted the spatial-patterns in RGC density changes, half-life and the extent of primary degeneration to be evaluated and summarised as colourmaps

## References

[bib1] Tham Y et al Am Acad Ophthalmol 2014; 121: 2081–2090. 10.1016/j.ophtha.2014.05.013 24974815

[bib2] Anderson DR et al Am J Ophthalmol 1998; 126: 487–497.9780093

[bib3] Chang EE et al Ophthalmology 2012; 119: 979–986. 22349567 10.1016/j.ophtha.2011.11.003 PMC3343191

[bib4] Titchenell PM et al Diabetes 2013; 62: 1808–1815. 23704522 10.2337/db12-1744 PMC3661651

[bib5] Russell WMS et al The principles of humane experimental technique. Methuen & Co. Ltd.: London, 1959.

[bib6] Cressey D Nature 2015; 520: 271. 25877180 10.1038/520271a

[bib7] Tannenbaum J et al J Am Assoc Lab Anim Sci 2015; 54: 120–132. 25836957 PMC4382615

[bib8] Guo L et al Cell Death Dis 2014; 5: e1460. 25321467 10.1038/cddis.2014.399 PMC4237238

[bib9] Davis BM et al Cell Death Discov 2016; 2: 16031. 27551521 10.1038/cddiscovery.2016.31 PMC4979431

[bib10] Levkovitch-Verbin H et al IOVS 2003; 44: 3388–3393. 10.1167/iovs.02-0646 12882786

[bib11] Morrison JC et al Exp Eye Res 1997; 64: 85–96. 9093024 10.1006/exer.1996.0184

[bib12] Li HY et al Neural Regen Res 2014; 9: 565–574. 25206855 10.4103/1673-5374.130093 PMC4146235

[bib13] Quigley H et al Ophthalmology 1988; 95: 357–363. 3174003 10.1016/s0161-6420(88)33176-3

[bib14] Morrison J et al Exp Eye Res 1995; 60: 127–135. 7781741 10.1016/s0014-4835(95)80002-6

[bib15] Guo L et al Curr Alzheimer Res 2010; 7: 3–14. 20205667 10.2174/156720510790274491

